# Highly Efficient Conversion of Greenhouse Gases Using a Quadruple Mixed Oxide-Supported Nickel Catalyst in Reforming Process

**DOI:** 10.1021/acs.iecr.3c02030

**Published:** 2023-10-02

**Authors:** Orrakanya Phichairatanaphong, Nevzat Yigit, Günther Rupprechter, Metta Chareonpanich, Waleeporn Donphai

**Affiliations:** † KU-Green Catalysts Group, Department of Chemical Engineering, Faculty of Engineering, Kasetsart University, Bangkok 10900, Thailand; ‡ Institute of Materials Chemistry, Vienna University of Technology, Getreidemarkt 9/BC/01, Vienna 1060, Austria; § Center for Advanced Studies in Nanotechnology for Chemical, Food and Agricultural Industries, KU Institute for Advanced Studies, Kasetsart University, Bangkok 10900, Thailand

## Abstract

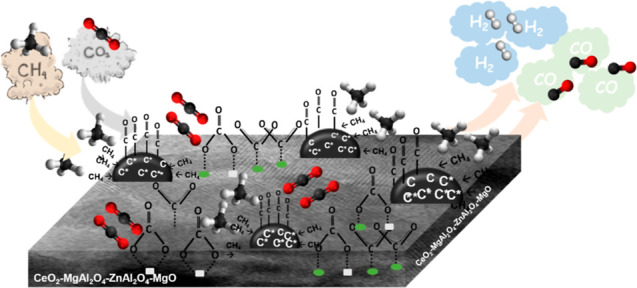

The greenhouse gas reduction as well as the utilization of more renewable and clean energy via a dry reforming reaction is of interest. The impact of a CeMgZnAl oxide quad-blend-supported Ni catalyst on performance and anticoking during dry reforming reactions at 700 °C was studied. A high Ce–Mg/Zn ratio, as seen in the CeMg0.5ZnAl-supported nickel catalyst, enhances lattice oxygen, and the presence of strong basic sites, along with the creation of the carbonate intermediate species, is accompanied by the production of gaseous CO through a gasification reaction between the carbon species and Ni-CO_ads-lin_ site. The phenomena caused the outstanding performance of the Ni/CeMg0.5ZnAl catalyst—CH_4_ (84%),CO_2_ (83%) conversions, and the H_2_/CO (0.80) ratio; moreover, its activity was also stable throughout 30 h.

## 1 Introduction

Global warming and the greenhouse effect are concerns for all countries worldwide, and it is essential to be aware of their impacts. The increasing emissions of the greenhouse gases directly contribute to rising global temperatures and extreme weather events. The main sources of the greenhouse gas emissions are electricity generation, transportation, industrial processes, and agricultural activities, with carbon dioxide (CO_2_) and methane (CH_4_) being the most significant contributors. Owing to the increasing demand for the utilization of the greenhouse gases, along with the growing interest in renewable and cleaner sources of energy, the process of reforming reaction, which involves combining CO_2_ and CH_4_, to produce the synthesis gas, a mixture of hydrogen (H_2_) and carbon monoxide (CO), has gained significant attention.^1–3^ The H_2_ obtained from this process can be utilized as a clean and sustainable energy source, while its reaction with CO via the Fischer–Tropsch process can generate high value-added hydrocarbons (olefin/paraffin) to serve as raw materials in petrochemical industries.^1–3^ The dry reforming reaction is a process that is endothermic in nature and necessitates high temperatures to be carried out successfully. To decrease the energy required for the reaction, active catalysts are necessary. Among the various catalysts investigated, nickel-based catalysts exhibit remarkable performance at moderate temperatures (500–600 °C) and low toxicity.^4–6^ However, the utilization of nickel catalysts is limited by their proneness to degradation caused by coking and the high-temperature sintering effect.

By improving the dispersion of the nickel metal, enhancing the surface basicity properties, and inhibiting coke deposition on the surface, the incorporation of a support significantly enhances both the activity and stability of the catalyst. A variety of metal oxides, including cerium oxide (CeO_2_),^7^ magnesium oxide (MgO),^8^ alumina (Al_2_O_3_),^9^ zinc oxide (ZnO), magnesium aluminate (MgAl_2_O_4_), and zinc aluminate (ZnAl_2_O_4_),^10^ have been employed as supports in the dry reforming reaction. Furthermore, mixed metal oxides, for instance, CeO_2_–MgAl_2_O_4_, CeO_2_–ZnAl_2_O_4_, Al_2_O_3_–CeO_2_, Ce_1–*x*_Zr_*x*_O_2_, and MgO–CeO_2_, have also been utilized as supports in the dry reforming reaction due to their unique surface characteristics. The CeO_2_ surface comprised Ce^4+^ and Ce^3+^ species on its lattice site bounded by oxygen ions, leading to the presence of oxygen vacancies, which adsorbed the oxygen formed by the CO_2_ dissociation and helped reduce coke accumulation on the catalyst surface.^11–13^ Alkaline metal oxides, such as MgO, increase the basic surface properties, resulting in improved CO_2_ adsorption and coke resistance.^13^ ZnO promotes CO_2_ adsorption and dissociation, as well as nickel dispersion, leading to an increase in the activity and stability while simultaneously decreasing carbon formation through the reverse Boudouard reaction.^14,15^ Additionally, at high calcination temperatures, MgO and ZnO can form MgAl_2_O_4_ and ZnAl_2_O_4_ spinel structures, respectively. These spinel structures can inhibit the deposition of coke on the surface by means of their strong interaction with the nickel support.^10,16^ Although mixed metal oxide-supported nickel catalysts are extensively employed in dry reforming, additional investigation is required to improve the catalyst’s activity and stability.

Herein, the metal oxide quad-blend—Ce, Mg, Zn, and Al—is a new alternative beneficial for boosting the performance and lifetime of the nickel-based catalyst because of their synergistic effect. The mixed metal oxide support with different Ce, Mg, Zn, and Al ratios was synthesized using a soft template-assisted coprecipitation procedure. Essential insights into the catalyst activities were uncovered through the investigation of the characteristic details,—conversions, product formation, and stability in the dry reforming reaction of a metal oxide quad-blend-supported nickel catalyst—particularly via in situ diffuse reflectance infrared Fourier transform spectroscopy (DRIFTS). The outstanding high-performance CH_4_ and CO_2_ conversions, as well as H_2_/CO ratio, were demonstrated by the Ce–Mg-0.5Zn–Al-supported nickel catalyst combination according to the results. Furthermore, its activity was stable during the reaction time. The impact of the molar ratio of tetra-metal oxide on the catalyst’s activity enhancement and the prolongation of its anticoking properties is due to its influence on the oxygen defects, the capacity for CO_2_ adsorption and dissociation on the surface, and the active nickel stability.

## 2 Experimental Section

### 2.1 Information on the Chemicals and Reagents

All the reagents and materials were of analytical grade. These include Pluronic P123 (P123: EO_20_PO_70_EO_20_, Sigma-Aldrich), cerium nitrate hexahydrate [Ce(NO_3_)_3_·6H_2_O, Sigma-Aldrich], magnesium nitrate hexahydrate [Mg(NO_3_)_2_·6H_2_O, Daejung], zinc nitrate hexahydrate [Zn(NO_3_)_2_·6H_2_O, LOBA], aluminum nitrate nonahydrate [Al(NO_3_)_3_.9H_2_O, Alpha Chemika], nickel nitrate hexahydrate [Ni(NO_3_)_2_·6H_2_O, QREC], and sodium hydroxide (NaOH, Carlo Erba).

### 2.2 Metal Oxide Quad-Blend-Supported Nickel Catalyst Procedure

A CeMgZnAl oxide supported Ni catalyst was synthesized in two steps: first by preparing a mixed *z*CeyMg*x*ZnAl oxide support and then by loading Ni onto this *z*Ce*y*Mg*x*ZnAl support to form the Ni/*z*CeyMg*x*ZnAl catalyst. The support was synthesized through a soft Pluronic P123 template-assisted coprecipitation method. The Ce, Mg, Al, and Zn metal precursors were obtained from cerium nitrate hexahydrate, magnesium nitrate hexahydrate, aluminum monohydrate, and zinc nitrate hexahydrate, respectively. The desired molar metal precursors (Pluronic P123/metal ions = 0.01) were added to the Pluronic P123 solution under stirring conditions until complete dissolution was achieved. Then, the pH was adjusted to 10.5 using NaOH (conc. One M) and kept under stirring conditions for 50 min. Subsequently, the obtained solution was transferred in an autoclave; the hydrothermal treatment process was carried out at 80 °C and held for 24 h. The solid was collected via the filtration–washing process, dried, and then calcined at 750 °C for a holding time of 4 h in air to yield the final product. The supports were denoted as *z*Ce*y*Mg*x*ZnAl, where *z*, *y*, and *x* were the molar ratios of Ce, Mg, and Zn, respectively. For comparison, the CeMgAl, CeZnAl, and Ce supports were prepared using the following method with variations that excluded Zn, Mg, or both Zn and Mg precursors, respectively.

The Ni/*z*Ce*y*Mg*x*ZnAl catalyst with 10 wt % Ni was prepared using an impregnation method using nickel nitrate hexahydrate as the nickel source. Elaboration of the methodology, alongside the structural and surface chemical properties of the Ni/*z*Ce*y*Mg*x*ZnAl catalyst characterized through diverse techniques, can be found in the Supporting Information.

### 2.3 Catalyst Test

The nickel loaded on a mixed metal oxide support with different molar ratios of metal (Ni/*z*Ce*y*Mg*x*ZnAl) was examined in a dry reforming reaction. The Ni/*z*Ce*y*Mg*x*ZnAl catalyst (0.1 g) positioned inside the Inconel tube reactor (3/8-in. outside diameter (O.D.)) was activated in the H_2_ atmosphere at 700 °C, holding for 1 h. Then, CH_4_ and CO_2_ gases were fed at 40 mL/min and a 1:1 molar ratio to the packed-bed reactor. The system was maintained under atmospheric pressure while being operated at a reaction temperature of 700 °C for a duration of 10 h. The conversion and product were determined through the analysis of the thermal conductivity detector gas chromatograph data, with Unibead-C serving as the packed column.

The calculations are performed using equations including CH_4_ and CO_2_ conversions and H_2_/CO molar ratio
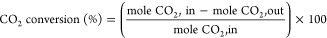

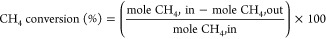

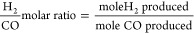


## 3 Results and Discussion

### 3.1 Interfacial Characteristics of Catalyst Structure and Surface

The Ni/CeMgAl and Ni/CeMg0.5ZnAl catalysts have a mesoporous structure with ink-bottle pores, confirmed by type IV isotherm with a H2 hysteresis loop^17^ (Figure S1A). The average pore sizes were 10.02 and 13.01 nm, respectively. The Ni/Ce, Ni/CeZnAl, and Ni/CeMgZnAl catalysts exhibited mesoporous structures containing slit-shaped pores with agglomerated plate-like particles, approved by the type IV isotherm—H3 hysteresis loop,^18^ which had an average pore size approximately 18.36, 15.71, and 22.14 nm, respectively (Figure S1B). According to Table S1, the Brunauer, Emmett, and Teller surface area of the Ni/CeMgAl and Ni/Ce catalysts was the highest (80 m^2^/g) and lowest (11 m^2^/g), respectively. The surface area of the Ni/*z*Ce*y*Mg*x*ZnAl catalysts falls in the range of 48–51 m^2^/g.

**Figure 1:**
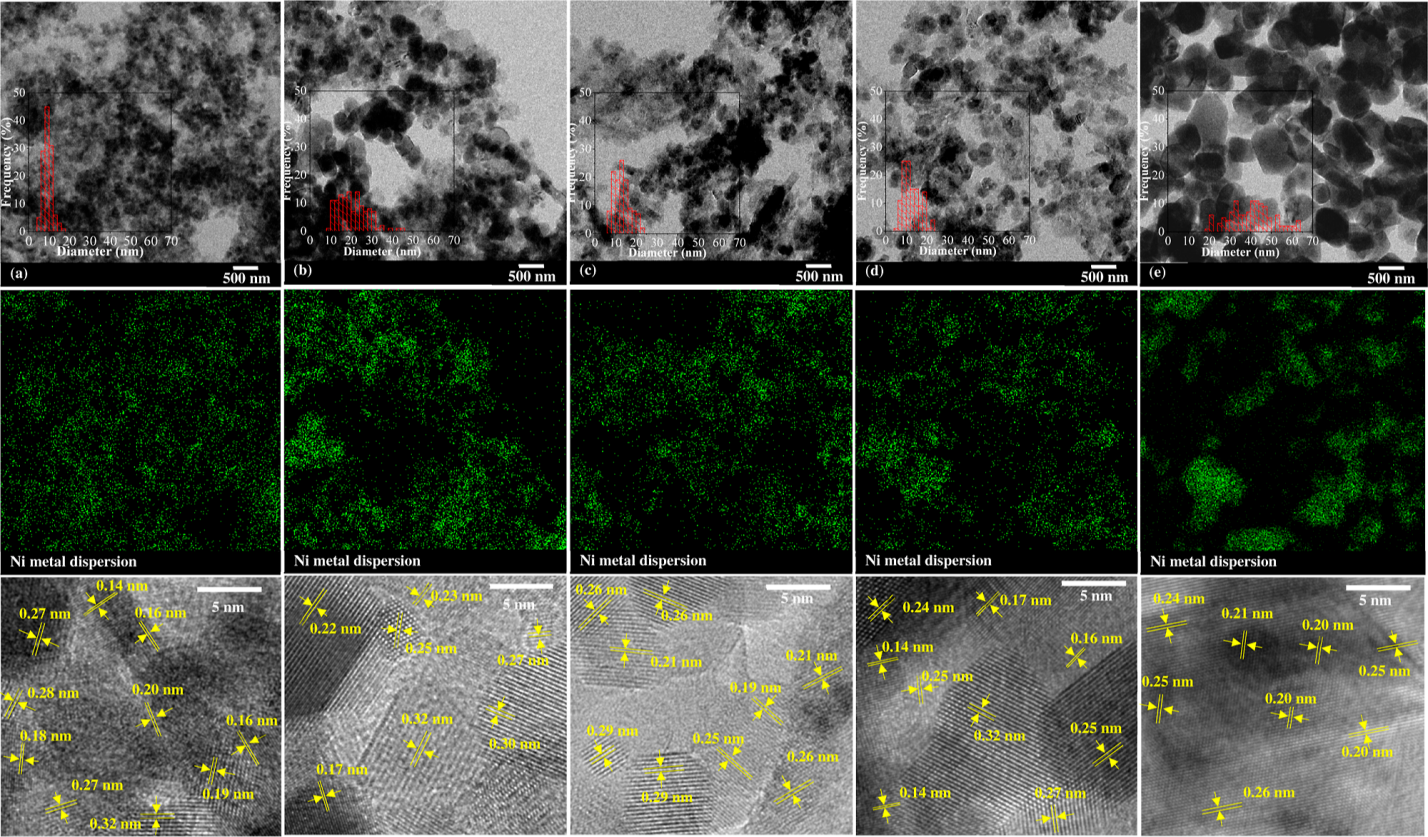
TEM images, nickel particle size distribution, and nickel energy-dispersive X-ray spectroscopy-mapping of the Ni/*z*Ce*y*Mg*x*ZnAl catalysts. (a) Ni/CeMgAl, (b) Ni/CeZnAl, (c) Ni/CeMgZnAl, (d) Ni/CeMg0.5ZnAl, and (e) Ni/Ce.

The Ni/*z*Ce*y*Mg*x*ZnAl catalyst structure and nickel dispersion were observed using the transmission electron microscopy (TEM) technique (Figure 1). The nanoparticles of the Ni/*z*Ce*y*Mg*x*ZnAl catalyst with different ratios of cerium, magnesium, and zinc were the agglomeration of sphere-like structures with well-proportioned geometrical aspect. Small NiO nanoparticles (Ni metal: green spot) were well dispersed on the *z*Ce*y*Mg*x*ZnAl supports in all catalysts. The particle sizes of NiO were ordered as follows: Ni/CeMgAl (∼9 nm), Ni/CeMg0.5ZnAl (∼10 nm), Ni/CeMgZnAl (∼13 nm), Ni/CeZnAl (∼20 nm), and Ni/Ce (∼42 nm). The lattice fringe analysis accomplished by high-resolution-TEM was used to clarify each type of metal oxide. The lattice fringes with interplanar spacings of 0.16–0.21, 0.24–0.28, 0.20–0.23, 0.23–0.27, and 0.16–0.18 nm corresponding to the NiO (111) plane, the CeO_2_ (111) plane, the MgO (111) plane, the ZnO (100) plane, and the MgAl_2_O_4_/ZnAl_2_O_4_ (440) planes, respectively, were found in all cases of catalysts. These interlayer spacings conformed to the X-ray diffraction (XRD) results. Moreover, the other metals including Ce, Mg, Zn, and Al were also well dispersed in the catalyst (Figure S2).

**Figure 2:**
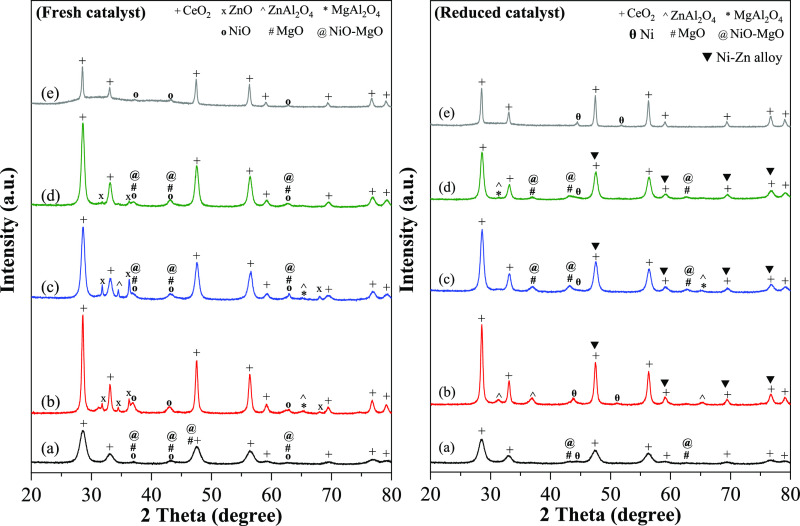
XRD of the fresh and reduced Ni/*z*Ce*y*Mg*x*ZnAl catalysts. (a) Ni/CeMgAl, (b) Ni/CeZnAl, (c) Ni/CeMgZnAl, (d) Ni/CeMg0.5ZnAl, and (e) Ni/Ce.

Figure 2A,B shows the crystallinities of nickel and mixed metal oxides of fresh and reduced catalysts, respectively. All fresh catalysts demonstrated the diffraction peaks at 2θ of 28.6, 33.3, 47.8, 56.4, 59.3, 69.4, 77.0, and 79.3°, corresponding to the existence of the CeO_2_ phase,^19^ and the diffraction peaks at 2θ of 37.0, 43.1, and 62.8° were attributed to MgO and NiO.^20^ The diffraction peaks at 2θ of 31.8, 34.5, 36.3, 63.0, and 68.1° confirmed the existence of ZnO phase^21^ found in the Ni/CeZnAl and Ni/*z*Ce*y*Mg*x*ZnAl catalysts. Under the calcination temperature at 750 °C, the nickel ion (Ni^2+^) could migrate to the surface MgO matrix to form the NiO–MgO solid solution on the Ni/CeMgAl and Ni/*z*Ce*y*Mg*x*ZnAl catalysts.^22^ The NiO–MgO solid solution peaks at 37.0, 43.1, and 62.8°, which are similar to the diffraction pattern of NiO and MgO,^22^ were detected in the Ni/CeMgAl and Ni/*z*Ce*y*Mg*x*ZnAl catalysts. The small diffraction peaks which were hardly noticeable at 2θ of 31.1 and 65.3° were matched to the MgAl_2_O_4_ and/or ZnAl_2_O_4_ phases, respectively.^21,23,24^

After the reduction process with H_2_ gas at 700 °C, the peak of the NiO–MgO solid solution, MgAl_2_O_4_, and/or ZnAl_2_O_4_ phases were still found in the reduced catalyst, but some diffraction peaks slightly changed compared to those of the fresh catalysts (Figure 2B). The diffraction peaks at 44° related to the metallic nickel phase^4^ were found in all the reduced catalysts. However, the diffraction peak of ZnO disappeared because the ZnO phase could react with NiO to form the Ni–Zn alloy phase during the reduction step,^25^ which was confirmed by the diffraction peaks at 43.0, 47.5, 59.0, 69.5, and 76.0° (the Ni–Zn alloy phase)^14,25^ found on the reduced catalysts. Although the peak positions of the Ni–Zn alloy were the same as the peak position of the CeO_2_ phase, this intensity of the reduced catalysts increased, implying the existence of the Ni–Zn alloy phase in the catalyst.

**Figure 3:**
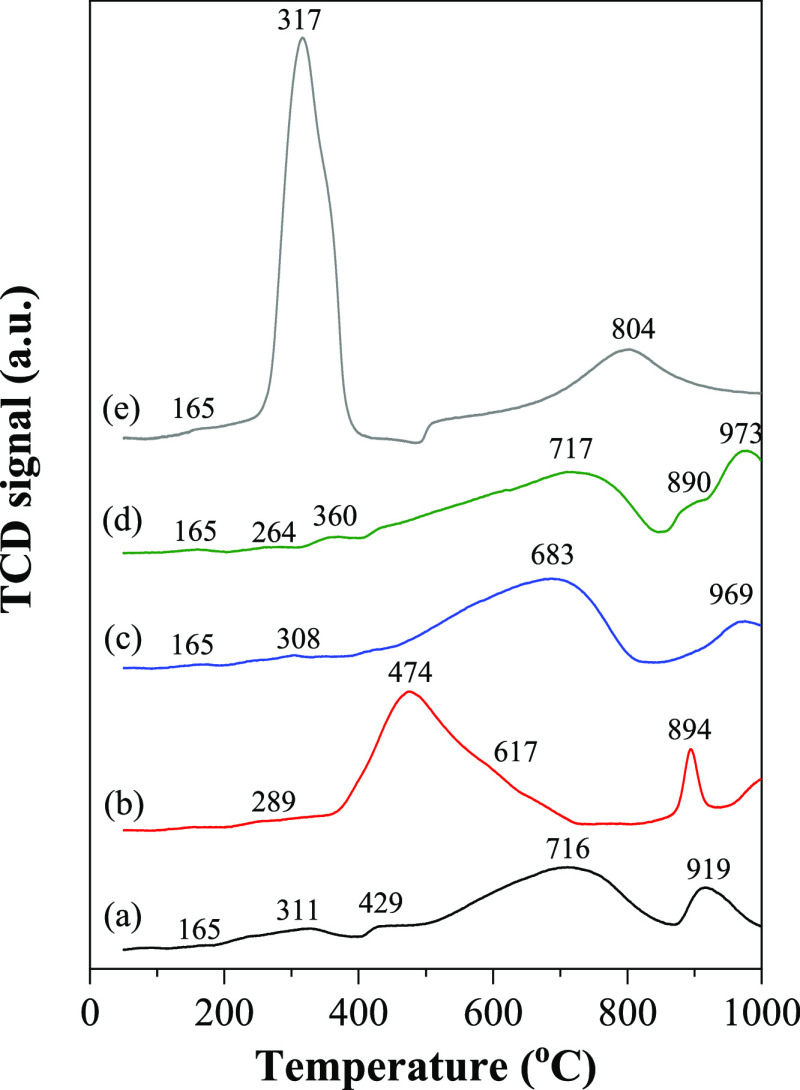
H_2_-TPR profiles of the Ni/*z*Ce*y*Mg*x*ZnAl catalysts. (a) Ni/CeMgAl, (b) Ni/CeZnAl, (c) Ni/CeMgZnAl, (d) Ni/CeMg0.5ZnAl, and (e) Ni/Ce.

The interaction behavior of Ni-mixed *z*Ce*y*Mg*x*ZnAl oxide examined by the H_2_-temperature-programmed reduction (TPR) profile is displayed in Figure 3. For the Ni/Ce catalyst, a small peak below 200 °C was correlated to the reduction of the surface-adsorbed oxygen species.^6^ The peak at 300–400 °C was related to the bulk NiO reduction.^10^ A temperature higher than 800 °C was due to the bulk CeO_2_ crystallite reduction.^26^ After the support was modified with Zn and Al, the Ni/CeZnAl catalyst showed a dominant peak centered at 474 °C, ascribed to the NiO reduction (NiO → Ni^0^),^10^ and the nickel reduction process shifted to a higher temperature. This shift implied a stronger interaction between the nickel and the CeZnAl support. The shoulder centered at 617 °C could be related to the NiO–ZnO coreduction.^14^

In the case of modification with Mg and Al, the Ni/CeMgAl catalyst displayed its main peak within the 500–850 °C temperature range. This peak was linked to two phenomena: the strong interaction of NiO with the surface, and the presence of the NiO–MgO solid solution.^22,23^

For the quadruple mixed oxide—Ni/CeMgZnAl catalysts—the H_2_-TPR profiles were analogous to those of the Ni/CeMgAl catalysts. However, the reduction temperature of the principal peak varied slightly based on the molar ratio of the Ce–Mg/Zn metals. Within the temperature ranges of 500–850 °C, the reduction was related to the presence of the NiO–MgO solid solution phase in the catalyst.^21^ As a result, the quadruple mixed oxide support helped improve the interaction between nickel and *z*Ce*y*Mg*x*ZnAl, further impacting the activity and stability of the dry reforming reaction.

**Figure 4:**
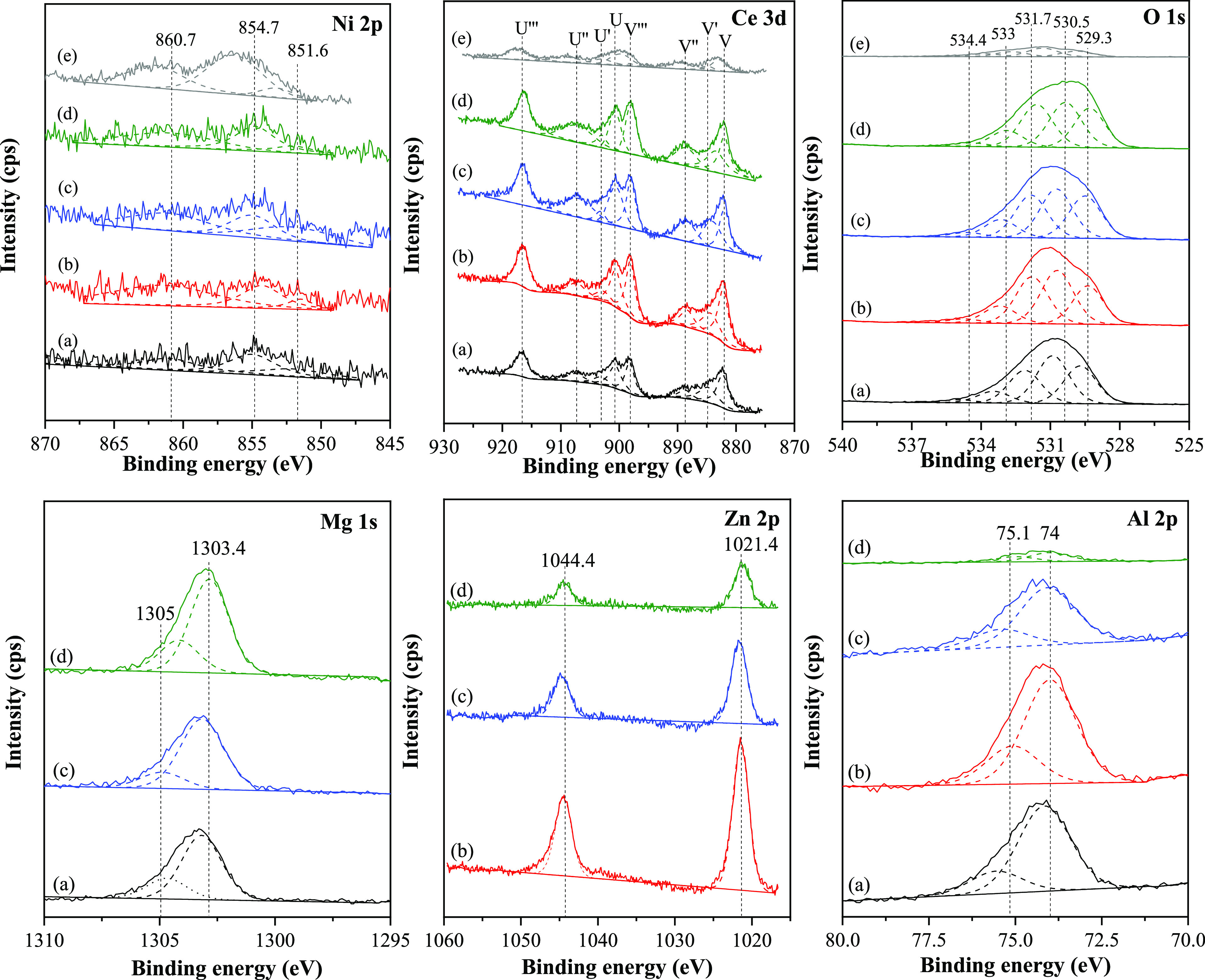
XPS spectra of the Ni 2p, Ce 3d, O 1s, Mg 1s, Zn 2p, and Al 2p of the reduced Ni/*z*Ce*y*Mg*x*ZnAl catalysts. (a) Ni/CeMgAl, (b) Ni/CeZnAl, (c) Ni/CeMgZnAl, (d) Ni/CeMg0.5ZnAl, and (e) Ni/Ce.

The XPS spectra verified the elemental chemical states in the reduced catalyst; all spectra were adjusted using the C 1s reference at a binding energy of 285 eV, as illustrated in Figure 4. The Ni 2p_3/2_ spectra could be separated into 3 peaks at 852, 855, and 861 eV assigned to the metallic nickel, Ni^2+^ phase, and the satellite of complex nickel, respectively.^6,27^ The peak intensity of the nickel oxide (Ni^2+^) phase was higher than that of the metallic nickel phase because of the occurring complex NiO in the NiO–MgO solid solution phase;^28^ meanwhile, the nickel oxide was partially reduced to form the metallic nickel at 700 °C, as assured by XRD and H_2_-TPR results.

The deconvoluted Ce 3d spectra of the reduced catalyst could indicate that the labels u and v correspond to Ce 3d_3/2_ and Ce 3d_5/2_ spin–orbit states, respectively. The peaks observed at 882 (*v*), 888 (*v″*), and 898 (*v‴*) were attributed to the Ce^4+^ (Ce 3d_3/2_) electronic states, whereas the peaks identified at 900 (*u*), 907 (*u″*), and 916 (*u‴*) were associated with Ce^4+^ (Ce 3d_5/2_) electronic states. The peak positions at 884 and 903 labeled as *u′* and *v′* were attributed to Ce^3+^.^6,29^ This confirmed the concurrent existence of Ce^3+^ and Ce^4+^ on the surface of the catalyst. The highest relative concentration of Ce^3+^ calculated from the deconvoluted peak area was found on the Ni/CeMg0.5ZnAl and Ni/CeMgAl catalysts (Table 1). A high amount of the Ce^3+^ species helps promote the oxygen vacancy/oxygen mobility by means of the ceria species shifting between Ce^4+^ and Ce^3+^.

**Table 1:** Active Nickel Area, Ce^3+^/Total Ce, Lattice/Vacancy Oxygen, and Basic Properties of the Catalysts

				basic site^c^ (μmol/g_cat_)			
catalyst	active nickel area^a^ (m^2^/g_metal_)	Ce^3+^/total Ce^b^ (%)	lattice/vacancy oxygen	weak	medium	strong	strong basic site (%)
Ni/CeMgAl	5.11	26.36	0.79	77.14	112.14	37.62	16.50
Ni/CeZnAl	2.41	22.39	0.67	105.60	65.42	31.39	15.50
Ni/CeMgZnAl	3.09	23.80	0.78	78.08	86.23	43.90	21.10
Ni/CeMg0.5ZnAl	3.48	26.37	0.83	101.63	79.23	42.41	19.00
Ni/Ce	n.d	15.32	0.66	29.53	30.68	9.78	13.90

a Calculated by CO pulse chemisorption.

b Calculated by XPS data.

c Calculated by CO_2_-TPD data.

The XPS O 1s core-level binding energies can be segregated into five peaks. The binding energy of 533 eV can be attributed to a hydroxide OH-type species, while the binding energy of 534.4 eV corresponds to the physically adsorbed oxygen species that forms as water due to the ambient moisture,^30^ and the binding energy of 531.7 eV ascribed to the surface chemisorbed species or the Ce^3+^ surface defects,^30,31^ at 530.5 eV (O_β_) indicated the surface adsorbed oxygen species, and that of 529.3 eV (O_α_) was related to lattice oxygen (O^2–^).^32,33^ As shown in Table 1, the highest and lowest lattice/vacancy oxygen ratio were established in the Ni/CeMg0.5ZnAl and Ni/CeZnAl catalysts, respectively. A higher lattice oxygen helps increase the methane oxidation rate and reacts with the carbonaceous intermediates and the solid carbon on the catalyst during the reaction.^34,35^

The Mg 1s, Zn 2p, and Al 2p XPS spectra of the reduced catalyst are shown in Figure 4. The Mg 1s spectra exhibited the binding energy at 1303.4 and 1305.1 eV which confirmed the presence of Mg^2+^ in the tetrahedral and octahedral sites, respectively.^36^ There are two types of binding energies detected at 1045 and 1022 eV, which are attributed to Zn 2p_1/2_ and Zn 2p_3/2_, respectively. These two energies have a difference of 23.0 eV, which suggests that there is Zn^2+^ present in the structure.^37^ The Al 2p spectra showed two peaks separated by a binding energy of 74 and 75.1 eV. The peak at 74 eV is related to Al^3+^ in a tetrahedral form, as seen in Al_2_O_3_. The peak at 75.1 eV corresponds to Al^3+^ occupying the octahedral sites in the spinel structures like MgAl_2_O_4_ and ZnAl_2_O_4_.^38^ The octahedral/tetrahedral ratio of the Ni/CeMg0.5ZnAl catalyst was greater than those of the other catalysts, demonstrating that this proportion of metal support helped to create more MgAl_2_O_4_ and ZnAl_2_O_4_ spinel structures.

Table 1 shows the results of analyzing the active nickel area of Ni/*z*Ce*y*Mg*x*ZnAl using the CO pulse chemisorption technique. The highest active nickel surface area was noticed in the Ni/CeMgAl catalyst followed by the Ni/CeMg0.5ZnAl, Ni/CeMgZnAl, and Ni/CeZnAl catalysts, respectively. This result implied that a higher catalyst surface area led to a higher active nickel dispersion on the surface.

**Figure 5:**
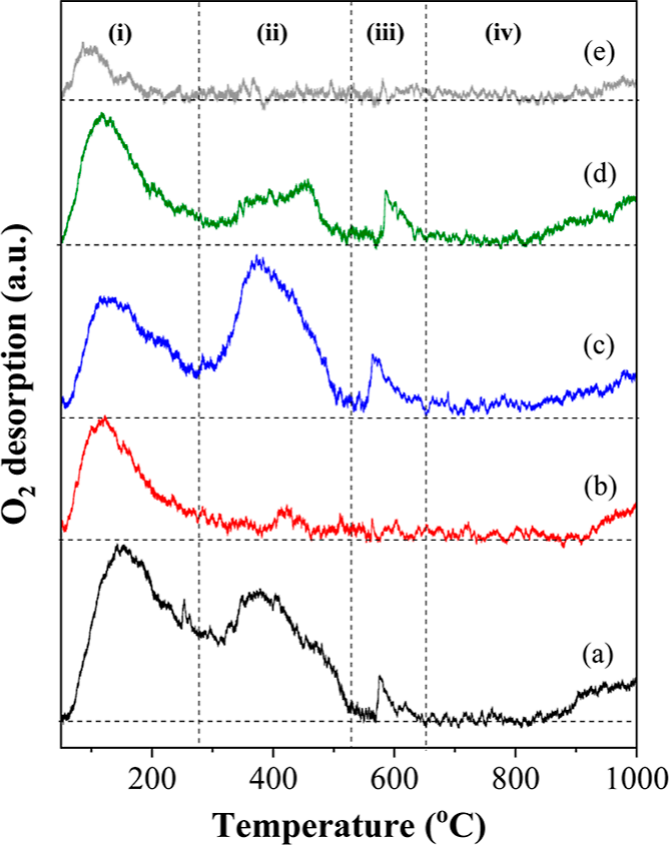
O_2_-TPD profiles of the Ni/*z*Ce*y*Mg*x*ZnAl catalysts. (a) Ni/CeMgAl, (b) Ni/CeZnAl, (c) Ni/CeMgZnAl, (d) Ni/CeMg0.5ZnAl, and (e) Ni/Ce.

To further analyze the distribution of the oxygen species on the Ni/*z*Ce*y*Mg*x*ZnAl catalyst surface, the O_2_-TPD technique was used to investigate the catalysts, and the O_2_-TPD profile is presented in Figure 5. The Ni/CeMgAl, Ni/CeMgZnAl, and Ni/CeMg0.5ZnAl catalysts exhibited four apparent peaks: (i) the desorption peak at 100–150 °C related to the physiosorbed oxygen or oxygen weakly bonded to the surface, (ii) the desorption peak at 370–400 °C, which corresponded to the chemisorbed oxygen or oxygen vacancy or oxygen mobility, (iii) the desorption peak at 550–650 °C related to the surface lattice oxygen, and (iv) the peak above 700 °C, which was assigned to the lattice oxygen from the bulk.^39–41^ While the Ni/CeZnAl and Ni/Ce catalysts showed two desorption peaks in regions (i) and (iv), this implied that the physisorbed oxygen (or oxygen weakly bonded to the surface) and lattice oxygen were found on the surfaces of both catalysts. As a result, the Ni/CeMgAl, Ni/CeMgZnAl, and Ni/CeMg0.5ZnAl catalysts have substantially higher oxygen mobility and more lattice oxygen on the surface compared with those of the Ni/CeZnAl and Ni/Ce catalysts. This is confirmed by the alteration in the amount of the desorbed oxygen species, which is in good agreement with the XPS results (Figure 4).

**Figure 6:**
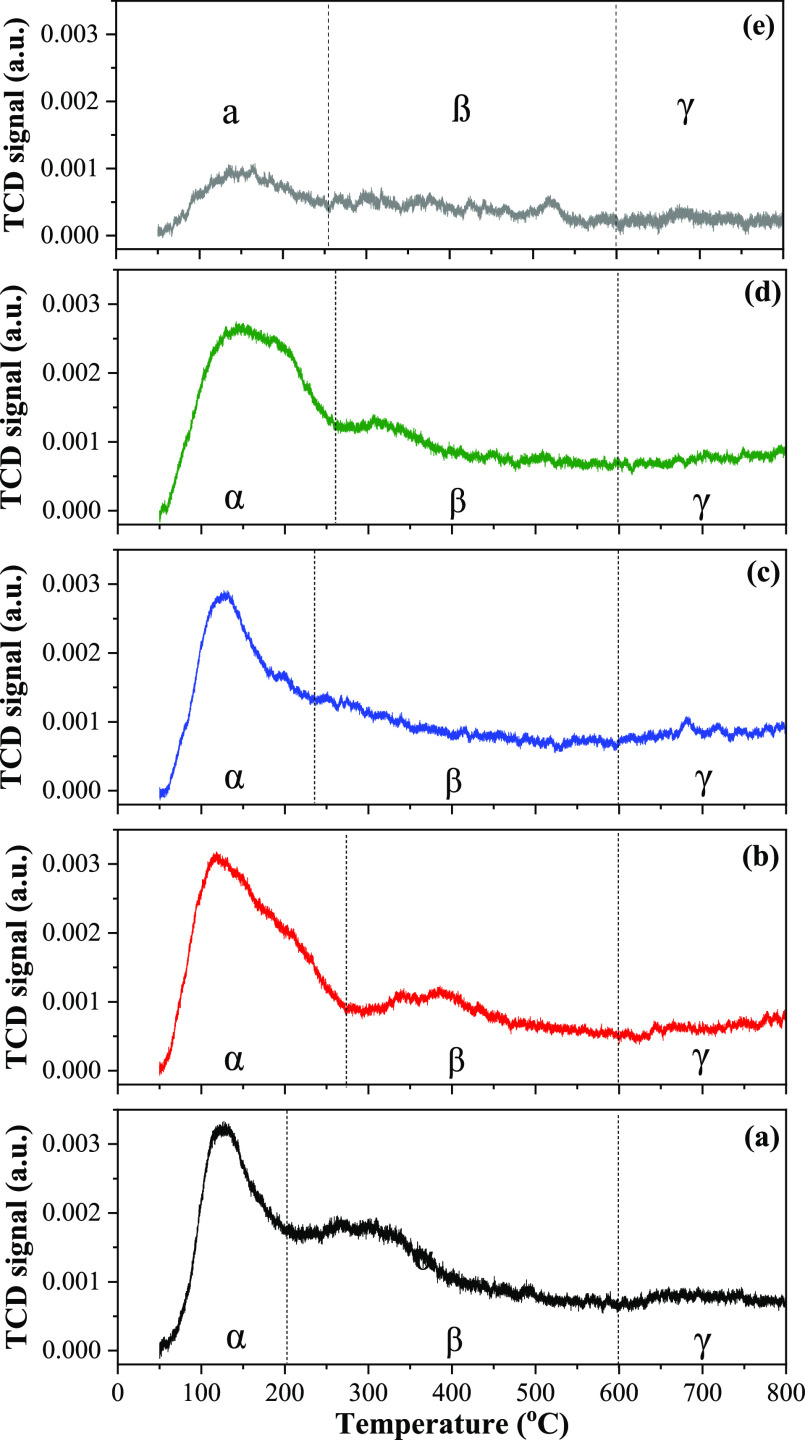
CO_2_-TPD profiles of the Ni/*z*Ce*y*Mg*x*ZnAl catalysts. (a) Ni/CeMgAl, (b) Ni/CeZnAl, (c) Ni/CeMgZnAl, (d) Ni/CeMg0.5ZnAl, and (e) Ni/Ce.

Figure 6 and Table 1 show the CO_2_-TPD profiles and the basic surface properties of the Ni/*z*Ce*y*Mg*x*ZnAl catalyst. All catalysts exhibited three desorption peaks including the alpha (α) zone, beta (β) zone, and gamma (γ) zone. The desorption temperature in alpha (α) zone (115–280 °C) could be attributed to the weak-strength basic site—weak Brønsted hydroxyl groups.^23,43^ The desorption in the beta (β) zone (280–600 °C) was ascribable to the medium-strength basic site, which is related to the formation of bidentate carbonate species on the metal–oxygen pairs and CO_2_ molecule surrounded with the low-coordination O^2–^ anions.^23,42,43^ The last desorption zone [gamma (γ)] at a temperature of around 600–800 °C comes from the strong-strength basic site—such as the monodentate carbonate or polydentate carbonate species.^44^ The deconvoluted CO_2_-TPD profiles expressed the basicity of the Ni/*z*Ce*y*Mg*x*ZnAl catalysts described in Table 1. The combination of Ce–Mg–Zn–Al oxide-supported nickel catalyst improved the CO_2_ adsorbed on a strong basic site at 700 °C, facilitating the dry reforming reaction.

### 3.2 Dry Reforming Activity

**Figure 7:**
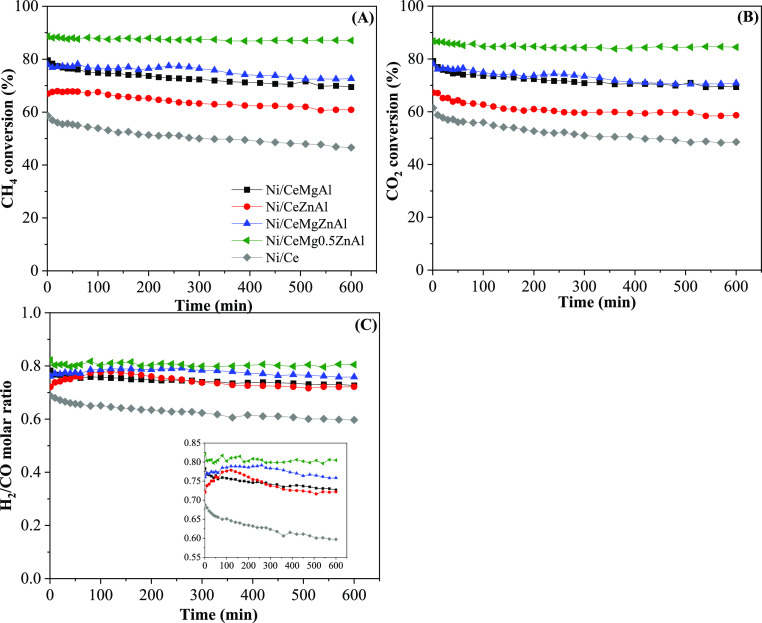
Performance of the Ni/*z*Ce*y*Mg*x*ZnAl catalysts with time on stream in dry reforming of methane reaction at 700 °C for 10 h. (A) CO_2_ conversion, (B) CH_4_ conversion, and (C) H_2_/CO.

The impact of the Ni/*z*Ce*y*Mg*x*ZnAl catalysts with varying Ce, Mg, and Zn molar ratios was investigated in a fixed-bed reactor over a 10 h period at a temperature of 700 °C during dry reforming reactions, as shown in Figure 7A–C. As the catalyst was activated at 700 °C under H_2_, the active nickel phase existed as both the metallic nickel (Ni^0^) and the NiO–MgO solid phase, as approved by the H_2_-TPR and XRD analysis. The Ni/CeZnAl catalyst had the lowest conversion and H_2_ and CO products, and the activity of the Ni/CeZnAl catalyst continuously lessened with increasing reaction time. For the Ni/*z*Ce*y*Mg*x*ZnAl catalysts, the proportion of Ce, Mg, and Zn had an effect on efficiency and stability during the dry reforming process. The Ni/CeMg0.5ZnAl catalyst demonstrated the greatest CH_4_ and CO_2_ conversions and the highest H_2_ to CO product ratios compared to other catalysts. Nevertheless, all catalysts exhibited a H_2_/CO ratio lower than one, attributable to the occurrence of the reverse water–gas shift reaction.^45^ Furthermore, the Ni/Ce catalyst was tested in a dry reforming reaction, and its activity and stability were compared to those of other Ni/*z*Ce*y*Mg*x*ZnAl catalysts. It was found that all Ni/*z*Ce*y*Mg*x*ZnAl catalysts exhibited significantly higher conversions and an improved H_2_/CO product ratio, as well as better stability during the time on stream. This could indicate that the CeMgZnAl-supported nickel catalysts with the coexistence of CeO_2_ and NiO–MgO solid solution—along with the MgAl_2_O_4_–ZnAl_2_O_4_ spinel structure—helps enhance and promote the dry reforming reaction. This composition could enhance the interaction between the Ni support, thereby enhancing the stability of the active nickel sites during the reaction. In addition, the altered surface basic sites become more favorable for CO_2_ adsorption, an important factor that enhances the reaction, especially in comparison to pure CeO_2_.

**Figure 8:**
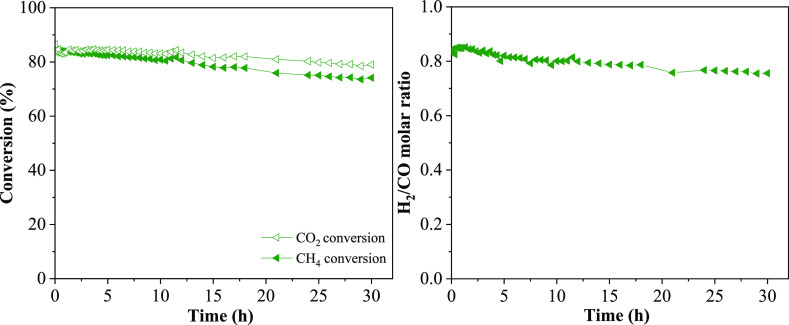
Performance of the Ni/CeMg0.5ZnAl catalysts with time on stream in dry reforming of the methane reaction at 700 °C for 30 h.

The CH_4_ and CO_2_ deactivation degrees expressed the stability of the catalyst throughout the time on stream for 10 h and are presented in Table 2. A lower deactivation degree is related to higher catalyst stability. The Ni/CeMg0.5ZnAl catalyst has the lowest CH_4_ and CO_2_ deactivation degrees approximately 7.62–25.62 times and 8.17–17.5 times lower than those of the other catalysts, respectively, implying that its activity was stable during the reaction. To further evaluate the activity and stability of the Ni/CeMg0.5ZnAl catalyst, a dry reforming reaction was conducted at 700 °C for 30 h; the performance is presented in Figure 8. The activity, in terms of CH_4_ and CO_2_ conversions using the Ni/CeMg0.5ZnAl catalyst, remained stable for 12 h and then slightly decreased. Moreover, a H_2_/CO molar ratio of approximately 0.8 was also stable throughout the time on stream. This implies that the proportion of CeMg0.5ZnAl in the nickel-supported catalyst has outstanding capabilities for converting CO_2_ and CH_4_ into syngas via the reforming process.

**Table 2:** Deactivation Degree of CH_4_ and CO_2_ and the Coke Deposition Rate of the Used Catalysts after Dry Reforming at 700 °C for 10 h

catalysts	CH_4_ deactivation degree^a^	CO_2_ deactivation degree^a^	coke deposition rate^b^ (g_carbon_/h. g_cat_)
Ni/CeMgAl	0.115	0.124	0.0011
Ni/CeZnAl	0.091	0.129	0.0140
Ni/CeMgZnAl	0.061	0.098	0.0125
Ni/CeMg0.5ZnAl	0.008	0.012	0.0074
Ni/Ce	0.205	0.210	0.0068

a Calculated as the deactivation degree = (CH_4_ or CO_2_ conversion at 1 min – CH_4_ or CO_2_ conversion at 10 h)/CH_4_ or CO_2_ conversion at 1 min.

b Calculated by Temperature Programmed Oxidation (TPO) data.

A high Ce–Mg/Zn ratio of tetra Ce, Mg, Zn, and Al-supported nickel catalyst not only elevates the efficacy in terms of conversion and H_2_/CO product ratio but also strengthens the stability of the catalyst throughout the reaction. A comparison of our catalyst’s performance compared with those reported in other research^10,13,24,27,46–48^ with different reaction conditions including the amount of catalyst and Ni loading, the reaction temperatures, and the total flow rate is provided in Table 3. The Ni/CeMg0.5ZnAl catalyst, functioning with a reduced catalyst quantity (0.1), a lower Ni loading (10 wt %), and a moderate reaction temperature (700 °C), continued to display superior activity, as evidenced by enhanced conversion and H_2_/CO ratios.

**Table 3:** Catalytic Performance of Ni/CeMg0.5ZnAl Compared with the Other Catalysts in Dry Reforming Reaction

	reaction conditions							
catalysts	amount of catalyst (g)	weight of Ni (wt %)	reaction temperature (°C)	total flow rate (mL/min)	CH_4_ conversion (%)	CO_2_ conversion (%)	H_2_/CO	refs
Ni/CeMg0.5ZnAl	0.1	10	700	40	88.6	86.7	0.82	this work
Ni-DLH	n.d	20	700	100	73	75	0.90	(44)
Ni/CeZr	0.4	5	700	160	55	67	0.87	(45)
Ni/MgO–Al_2_O_3_	n.d	10	700	n.d	75	80	n.d	(13)
Ni/CeO_2_	0.5	10	800	100	80	80	0.78	(28)
Ni/ZAO–Ce	0.2	12.5	700	60	76	88	0.98	(10)
Ni/CeO_2_–MgAl_2_O_4_	n.d	5	700	n.d	76	83	n.d	(24)
Ni–MgO–CeO_2_	0.015	15	800	405	69	71	0.94	(46)

### 3.3 Characteristics of the Used Ni/*z*Ce*y*Mg*x*ZnAl Catalysts after the Reaction

**Figure 9:**
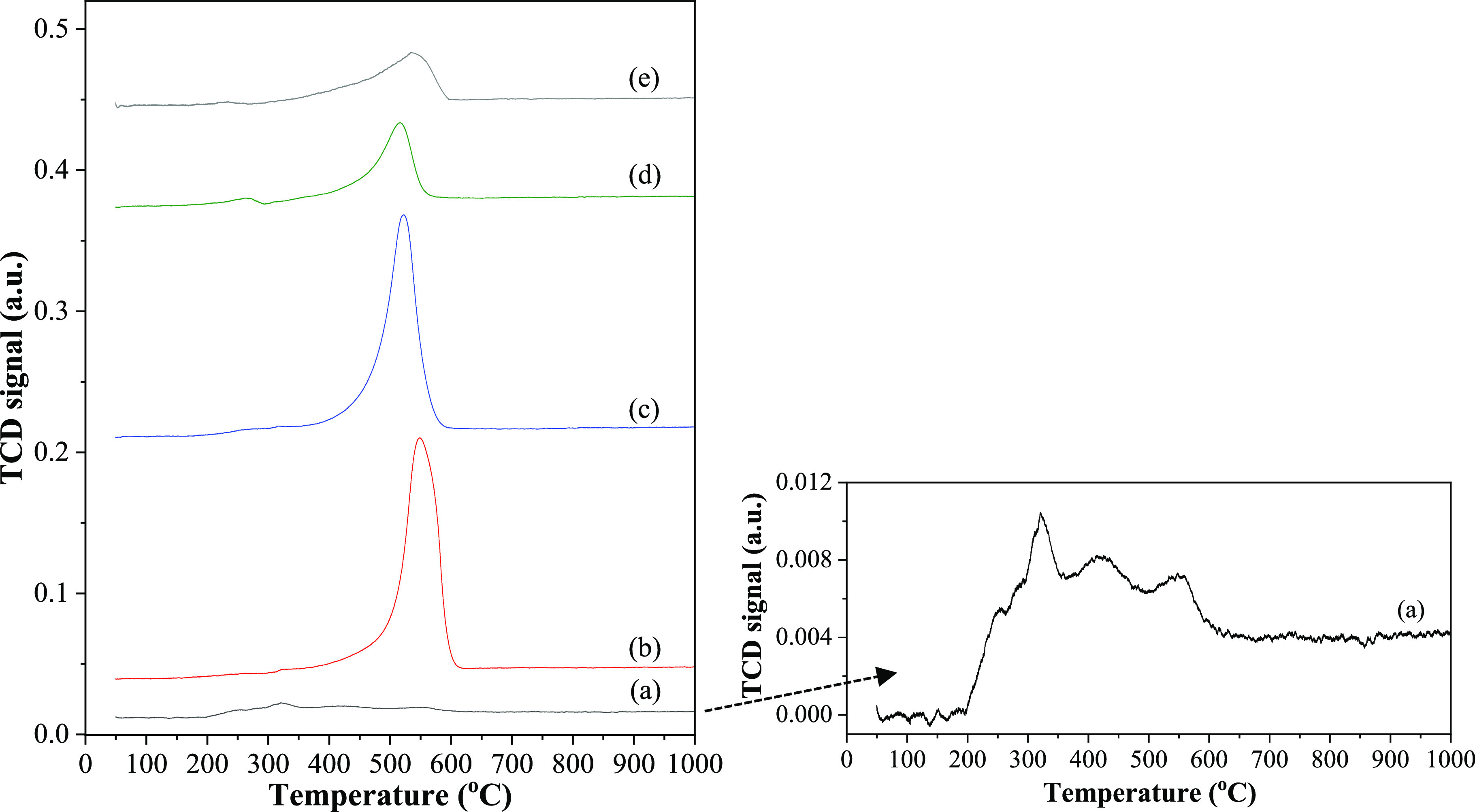
O_2_-TPO profiles of the Ni/*z*Ce*y*Mg*x*ZnAl catalysts after dry reforming at 700 °C for 10 h. (a) Ni/CeMgAl, (b) Ni/CeZnAl, (c) Ni/CeMgZnAl, (d) Ni/CeMg0.5ZnAl, and (e) Ni/Ce.

The coke accumulated on the catalyst surface after the reaction is analyzed by the O_2_-TPO technique; the TPO profile is shown in Figure 9. The oxidation peak corresponded to the nature of the catalyst and the carbon allotrope on the used catalyst. The oxidation temperature below 400 °C was related to the combustion of amorphous carbon.^49^ The high temperature in the range of 400–700 °C was attributed to the graphitic carbon/carbon nanotube.^49^ The coke deposition rate of the catalyst is presented in Table 2. The lowest amount of coke accumulated was noticed in the Ni/CeMgAl catalyst, followed by the Ni/CeMg0.5ZnAl catalyst. The decreasing coke formation was due to the coexistence of the redox property of CeO_2_ and the existence of the MgAl_2_O_4_ spinel and the NiO–MgO solid solution structures. Moreover, high lattice oxygen on the surface of the Ni/CeMg0.5ZnAl catalyst facilitated the decrease of carbon deposited by interacting with the adsorbed carbonaceous types on the surface.^34,35^

**Figure 10:**
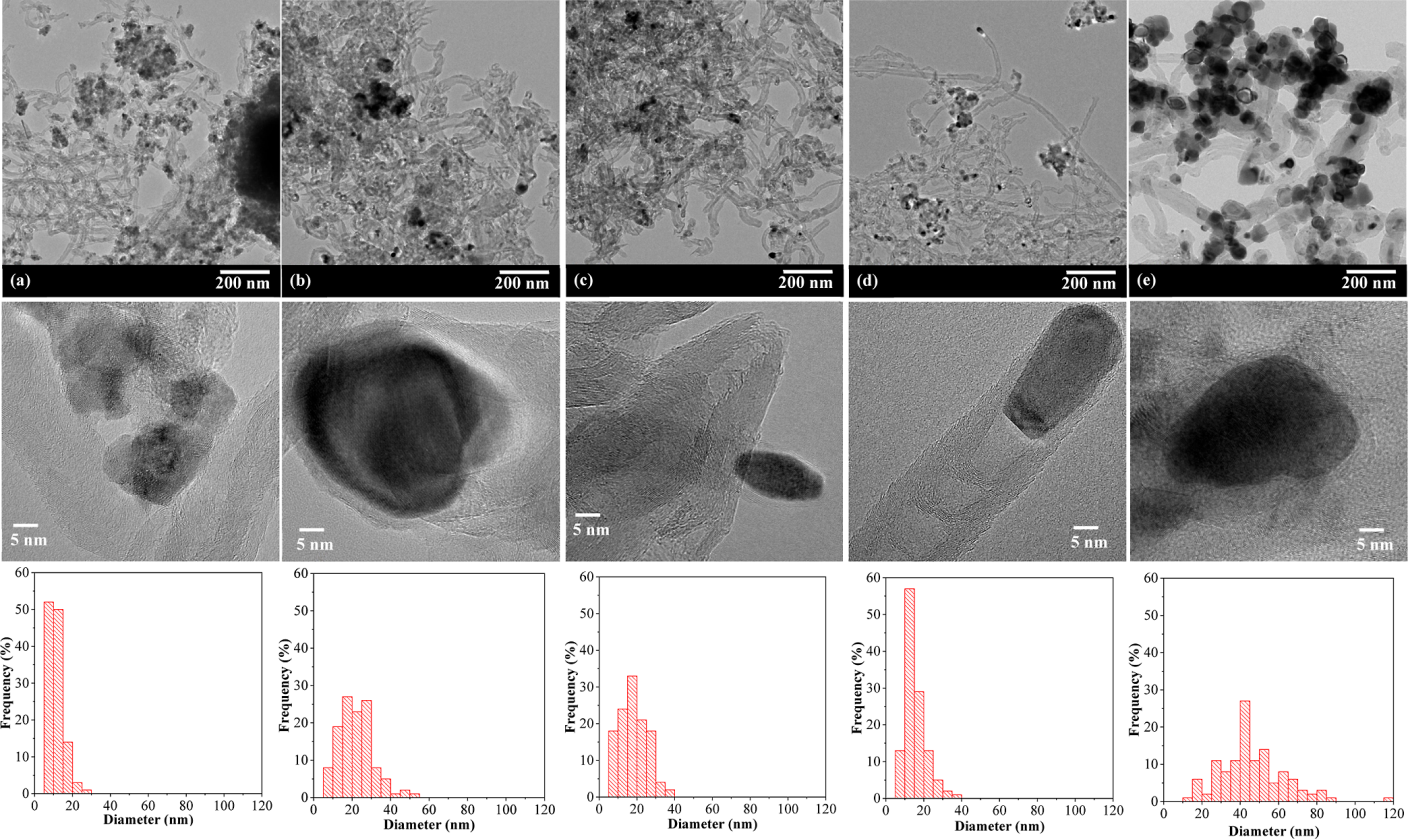
TEM images and histogram distribution of the used Ni/*z*Ce*y*Mg*x*ZnAl catalysts after dry reforming reaction. (a) Ni/CeMgAl, (b) Ni/CeZnAl, (c) Ni/CeMgZnAl, (d) Ni/CeMg0.5ZnAl, and (e) Ni/Ce.

Figure 10 shows the carbon/coke allotrope found on the catalyst using TEM. All the used catalysts were found to have a carbon nanotube (CNT) structure. However, CNTs of smaller diameter grew on the Ni/CeMgAl and Ni/CeMg0.5ZnAl catalysts, whereas those of a larger diameter size grew on the Ni/CeZnAl catalyst (as clearly seen in the histogram of Figure 10). The CNT size depended on the size of the active metal located on the support. It was clearly seen that a bigger nickel cluster size was located on the CeZnAl support, while smaller sizes were found on CeMgAl and CeMg0.5ZnAl supports because the nickel that interacted with the CeZnAl support was weaker than those of the other supports (consistent with H_2_-TPR result); therefore, the active nickel was easily agglomerative during the dry reforming reaction.

**Figure 11:**
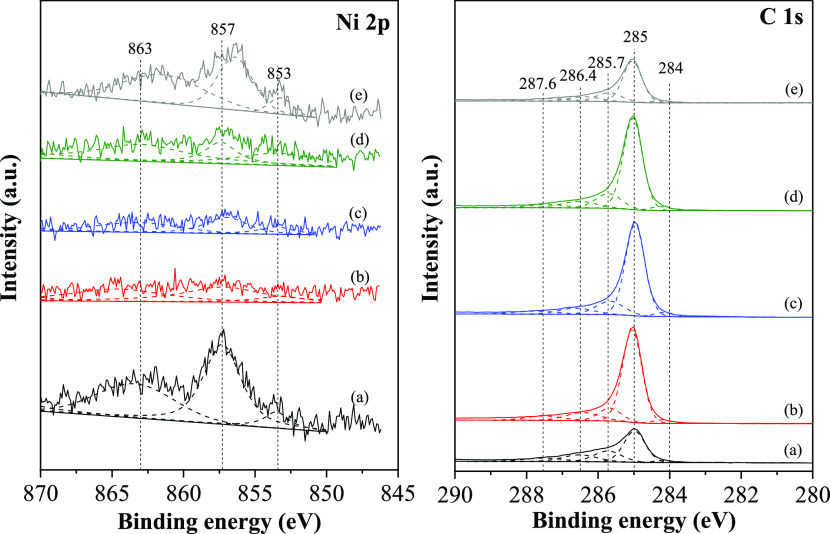
XPS spectra of Ni 2p and C 1s of the Ni/*z*Ce*y*Mg*x*ZnAl catalysts after dry reforming at 700 °C for 10 h. (a) Ni/CeMgAl, (b) Ni/CeZnAl, (c) Ni/CeMgZnAl, (d) Ni/CeMg0.5ZnAl, and (e) Ni/Ce.

The oxidation levels of nickel (Ni) and carbon (C) species of used catalysts after the reaction were studied using the XPS method, as demonstrated in Figure 11A,B, respectively. The Ni 2p_3/2_ binding energy peak of the used catalyst could be deconvoluted into three peaks of 853.2, 857.2, and 863.3 which were assigned to the metallic nickel, Ni^2+^ state, and the satellite peaks of complex nickel, respectively.^6,27^ The oxide quad-blend Ce–Mg–Zn–Al support could help stabilize the active metallic nickel, especially with the composition of Ce–Mg-0.5Zn–Al-supported nickel catalyst having the highest metallic nickel intensity, resulting in the Ni/CeMg0.5ZnAl catalyst showing the highest conversion, as well as better catalyst stability during the reaction. The C 1s spectra, accounting for the spin–orbit coupling, can be represented by five distinct peaks at 284.2, 285.0, 285.7, 286.4, and 287.6 eV, which correspond to the carbide phase, sp^2^-carbon (C=C), sp^3^-carbon (C–C), C–O, and C=O functional groups, respectively.^50^ The concentration of sp^2^-carbon and sp^3^-carbon hybridization and the relative sp^2^/sp^3^ intensity ratio are evaluated from the XPS measurements, as shown in Table S2. The structure of coke deposited on the catalyst surface consisted of sp^2^- and sp^3^-hybridization: a higher amount of coke deposition contained a higher carbon structure of sp^2^-hybridization.

### 3.4 Proposed Mechanism of Ni/*z*Ce*y*Mg*x*ZnAl Catalyst for Dry Reforming Reaction

**Figure 12:**
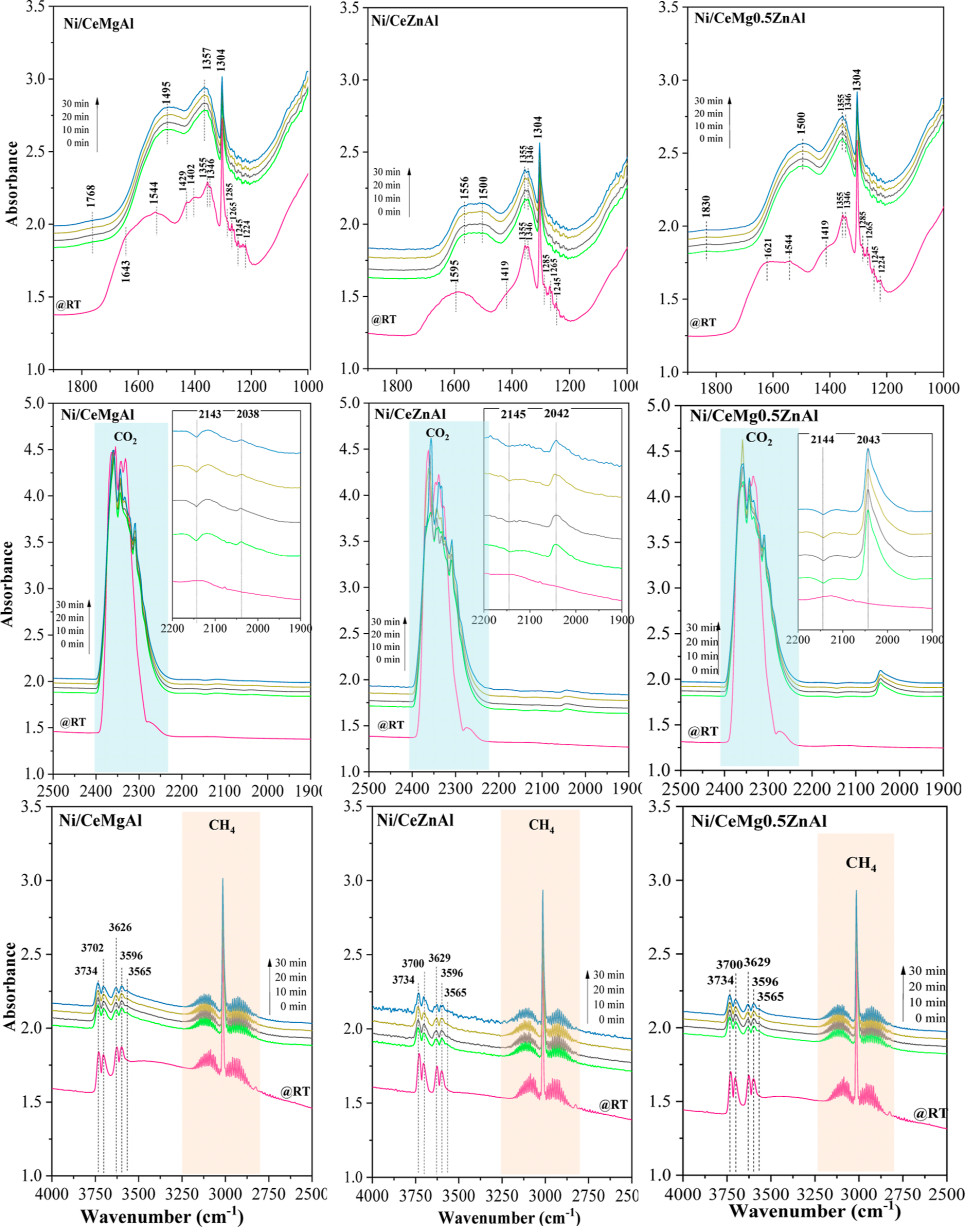
DRIFTS spectra obtained during dry reforming at 450 °C in the region of 1000–1900, 1900–2500 , and 2500–4000 cm^–1^ of catalysts.

To examine the adsorption of CH_4_ and CO_2_ on the surface of the catalyst and suggest the mechanism of the Ni/*z*Ce*y*Mg*x*ZnAl catalyst in the CO_2_ reforming of CH_4_ reaction, in situ DRIFTS was conducted under alternatively flowing equivalent volume of CH_4_ and CO_2_ at 450 °C. The DRIFTS spectra of the Ni/CeMgAl, Ni/CeZnAl, and Ni/CeMg0.5ZnAl catalysts divided into three segments of 1000–1900, 1900–2500, and 2500–4000 cm^–1^ at room temperature and 450 °C are displayed in Figure 12.

In the region of 1000–1900 cm^–1^, at room temperature, the band at 1304 cm^–1^ was assigned to the gas phase of CH_4_.^51^ The peaks at 1643, 1621, 1429 cm^−1^, and within the range of 1224–1285 cm^−1^ correspond to the bidentate carbonate species. Meanwhile, the peaks at 1346, 1355, and 1544 cm^−1^ are assigned to the monodentate carbonate species, and the peak at 1402 cm^−1^ relates to the bicarbonate species.^52–54^ After the reaction temperature raised to 450 °C, the peak intensities of the bidentate carbonate species significantly decreased, and some peaks of the monodentate carbonate species disappeared yet appeared at wavenumbers of 1556, 1355–1357, and 1346 cm^–1^. The polydentate carbonate species at the peak of 1495–1500 cm^–1^ was observed on the catalysts. Moreover, at 450 °C, the bridged carbonate species (1768 cm^–1^)^55^ formed on the Ni/CeMgAl surface due to its strong-strength basic sites of the Ni/CeMgAl catalyst resulted in strong CO_2_ adsorption on the catalyst surface. A new band at 1830 cm^–1^ related to the bridged CO species adsorbed on the Ni sites was found in the Ni/CeMg0.5ZnAl surface, indicating that the CO_2_ dissociative adsorption occurred on the Ni sites.^54^

In the region of 2500–1900 cm^–1^_,_ the high-intensity peak at 2360–2356 cm^–1^ corresponded to the CO_2_ gas.^56^ After the system was heated to 450 °C, the vibration band at 2143 cm^–1^ appeared because CH_4_ reacted with CO_2_ gases to produce the CO gas. Moreover, all the catalysts displaying a peak in the range of 2038–2043 cm^–1^ was the characteristic of the coordinated CO linearly adsorbed on the metal site;^51,57^ especially, the Ni/CeMg0.5ZnAl catalyst has the highest peak intensity of linear carbonyl species. It could explain that the surface property of the Ni/CeMg0.5ZnAl catalyst favored CO adsorption on the catalyst surface, and this phenomenon resulted in a low coke formation on the catalyst during the reaction (as confirmed by the TPO technique).

In the region of 4000–2500 cm^–1^, the vibrational band at 3016 cm^–1^ related to the CH_4_ gas phase^58^ was observed in all catalysts at room temperature; this peak intensity decreased after the temperature increased. The bands within the range of 3560–3740 cm^–1^ were associated with the surface hydroxyl groups (O–H), which constituted numerous peaks of surface hydroxyl groups, indicating the presence of various coordinated hydroxyl groups formed on the catalyst surface. The bands at 3702 and 3629 cm^–1^ were attributed to the monodentate bicarbonate and bidentate bicarbonate species, while the peaks at 3596 and 3567 cm^–1^ corresponded to the interacting hydroxyl groups of the formed bicarbonates.^59^ Furthermore, these peak intensities decreased with increasing temperature because the hydroxyl groups could promote the carbonaceous species oxidation by means of diffusion on the metal surface.

**Figure 13:**
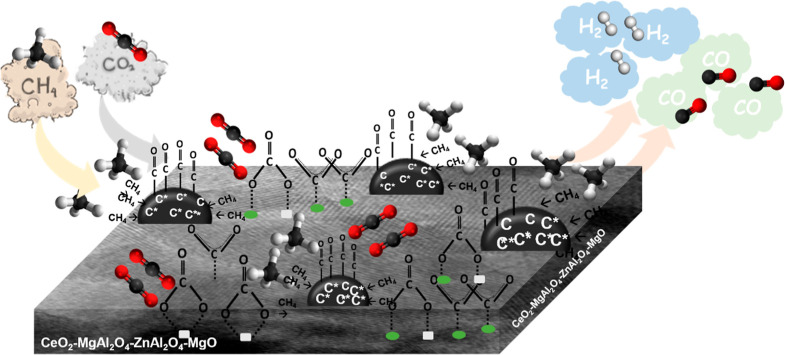
Proposed mechanism of the tetra Ce–Mg–Zn–Al oxide-supported nickel catalyst in the dry reforming reaction.

Regarding the characterization results, the possible and simplified mechanism of oxide quad-blend Ce–Mg–Zn–Al-supported nickel catalyst in dry reforming reaction could be proposed as presented in Figure 13. The surface of the Ni/CeMg0.5ZnAl catalyst features active Ni metal supported by a coexistence of CeO_2_, NiO−MgO solid solution, and a MgAl_2_O_4_–ZnAl_2_O_4_ spinel structure. This configuration moderates the surface basicity by reducing the presence of strong basic sites compared to the tri-metal oxides (CeMgAl and CeZnAl). This makes it more conducive for CO_2_ adsorption and simultaneous dissociation on the tetra-metal oxide surface. The CH_4_ molecule was adsorbed onto the bound active Ni sites to generate the CH_x_^*^ and H* species, which subsequently decomposed into carbon species (C*) that were deposited onto the catalyst surface and H_2_. Simultaneously, the CO_2_ molecule was adsorbed onto the surface oxygen of the tetra Ce–Mg–Zn–Al oxide to generate the carbonate species, which included both monodentate and polydentate carbonate species. These intermediate species could further lead to the formation of the CO gas. Moreover, a higher peak intensity of CO linearly adsorbed metallic nickel carbonyl species (Ni–CO_ads-lin_) contributed to a higher CO gas formation by the gasification between the carbon species (C*) or the carbonaceous and Ni–CO_ads-lin_ site. Hence, the presence of a tetra Ce–Mg–Zn–Al-supported nickel catalyst not only improves the activity in terms of conversion and product yield but also enhances the catalyst stability during the reaction.

## 4 Conclusions

The impact of the nickel/mixed CeMgZnAl oxide catalyst on the performance and lifetime in the dry reforming reaction at 700 °C was investigated. The mixed Ce–Mg–Zn–Al oxide supports were synthesized using the one-pot soft-template-assisted coprecipitation and Pluronic P123 used as a template. The results showed that the proportion of the Ce–Mg-0.5Zn–Al-supported Ni catalyst exhibited outstanding activities and stability during the reaction time. This catalyst consisted of a coexisting CeO_2_ and NiO–MgO solid solution with MgAl_2_O_4_–ZnAl_2_O_4_ spinal structures at a high Ce–Mg/Zn ratio that promoted a lattice oxygen/oxygen vacancy, strong basic site, the strong nickel–support interaction, capacity of CO_2_ adsorption, and dissociation on the catalyst surface, consequently improving the catalyst activity and prolonging the anticoking. Hence, the design of the nickel-based supported tetra Ce–Mg–Zn–Al oxide catalyst has been considered for H_2_ and CO production and decreasing the main greenhouse gases simultaneously via the CO_2_ reforming of CH_4_.

## Supporting Information Available

Textural properties of the Ni/*z*Ce*y*Mg*x*ZnAl catalyst; fitting result of sp^2^-carbon and sp^3^-carbon hybridization; relative sp^2^/sp^3^ intensity ratio evaluated from XPS measurements of the used catalyst; N_2_ adsorption–desorption isotherms, pore size distributions of Ni/*z*Ce*y*Mg*x*ZnAl catalysts: (a) Ni/CeMgAl, (b) Ni/CeZnAl, (c) Ni/CeMgZnAl, (d) Ni/CeMg0.5ZnAl, (e) Ni/Ce; and EDS-mapping of Ce, Mg, Zn, and Al of the fresh catalysts: (a) Ni/CeMgAl, (b) Ni/CeZnAl, (c) Ni/CeMgZnAl, (d) Ni/CeMg0.5ZnAl, and (e) Ni/Ce (PDF)
