# Supplementary material for: Highly Efficient Conversion of Greenhouse Gases Using a Quadruple Mixed Oxide-Supported Nickel Catalyst in Reforming Process
Source: Ind Eng Chem Res. 2023 Oct 2;62(40):16254–67. doi: 10.1021/acs.iecr.3c02030 (PMC10571087; doi:10.1021/acs.iecr.3c02030)
Supplement: Supplementary file 1 — ie3c02030_si_001.pdf [file ie3c02030_si_001.pdf]

Supplementary Material

Highly efficient conversion of greenhouse gases using a quadruple mixed oxide supported nickel catalyst in reforming process

Orrakanya Phichairatanaphong[a], *Nevzat Yigit*[b], *Günther Rupprechter*[b], *Metta Chareonpanich*[a,c], *Waleeporn Donphai*[a,c,*]

[a] *KU-Green Catalysts Group, Department of Chemical Engineering, Faculty of Engineering, Kasetsart University, Bangkok 10900, Thailand*

[b] *Institute of Materials Chemistry, Vienna University of Technology, Getreidemarkt 9/BC/01, Vienna 1060, Austria*

[c] *Center for Advanced Studies in Nanotechnology for Chemical, Food and Agricultural Industries, KU Institute for Advanced Studies, Kasetsart University, Bangkok 10900, Thailand*

* Corresponding authors. Tel.: +66 2579 2083; Fax: +66 2561 4621.

E-mail address: fengwod@ku.ac.th (Waleeporn Donphai)

1. Experimental

1.1 Ni/zCeyMgxZnAl catalyst preparation

The amount of 10 wt.% Ni was loaded into zCeyMgxZnAl support by using the incipient wetness impregnation method. First, nickel nitrate hexahydrate ($Ni(NO_3)_2.6H_2O$) was dissolved in deionized water until the clear solution. Then the solution was dropped into the support, and the mixture was stirred at room temperature for 1 h. After that, this mixture was dried by microwave at 800 W for 1 min and calcined at 550^oC for 4 h with a heating rate of 5^oC/min.

1.2 Catalyst characterizations

The textural properties of zCeyMgxZnAl supports and Ni/zCeyMgxZnAl catalysts were examined by N_2 physisorption (3Flex Adsorption Analyzer, Micromeritics). The Brunauer–Emmett–Teller (BET) and Barrett–Joyner–Halenda (BJH) methods were used to calculate the specific surface area and pore size distributions, respectively.

The crystalline structures of fresh and reduced Ni/zCeyMgxZnAl catalysts were characterized using X-ray diffraction spectroscopy (D8 Advance, Bruker). This operated at 40 kV and 40 mA with monochromated Cu Kα radiation in 2θ range of 10-80°.

The fresh Ni/zCeyMgxZnAl catalyst structure and metal dispersion were detected by transmission electron microscopy (TEM: JEOL JEM2100 instrument) operated at 200 kV acceleration voltage.

The nickel-mixed oxide support interaction and reduction behavior were characterized using the H_2-temperature programmed reduction (H_2-TPR) method (Autochem II Chemisorption analyzer, Micromeritics). Each experiment, the 10% H_2 in Ar gas with a total flow rate of 30 ml/min was fed into the system, and the temperature raised to 1000^oC with 5^oC/min of heating

rate. The quantity of H_2 consumption was evaluated by using a thermal conductivity detector (TCD).

To further analyze the distribution of oxygen species on the Ni/zCeyMgxZnAl catalyst surface, the O_2-temperature-programmed desorption (O_2-TPD) technique (Autochem II Chemisorption analyzer: Micromeritics). First, the catalyst (0.1 g) was preheated at 300oC for 1 h with O_2 in He flow, then the system was cooled down to room temperature. After that, He gas was purged to the catalyst bed to remove physically adsorbed species. The O_2 desorption profile was investigated by increasing the temperature from 50 to 1000°C at a heating rate of 5°C/min, using a thermal conductivity detector (TCD).

The basic surface property of the catalyst was determined by using the CO_2-temperature-programmed desorption (CO_2-TPD) technique (Autochem II Chemisorption analyzer: Micromeritics). First, the catalyst (0.1 g) was activated under H_2 flow at 700°C with a heating rate of 5°C/min for 1 h, after that purging with argon (Ar) gas for 1 h to clean the surface of active metal. Then, the system temperature was cooled down to 50°C, and the 10% CO_2 in He gas with a flow rate of 50 ml/min was introduced for 1 h to reach adsorption equilibrium. After that, Ar gas was then purged to the catalyst bed to remove physically adsorbed species. The CO_2 desorption profile was investigated with increasing temperature from 50 to 1000 °C with a heating rate of 5 °C/min using a thermal conductivity detector (TCD).

The oxidation state of each metal of the reduced and used Ni/zCeyMgxZnAl catalyst was examined by X-ray photoelectron spectroscopy (XPS) technique (AXIS Ultra DLD). The C 1s at 284 eV was the reference binding energy peak.

The active Ni surface area on the catalyst was determined by using the CO pulse chemisorption technique (Micromeritics: Autochem II Chemisorption analyzer). The catalyst

was reduced with H_2 gas at 700°C for 1 h. After that, the Ar gas was purged through the reduced catalyst for 1 h. The CO chemisorption was operated at 50°C by CO-pulse, and the amount of CO adsorbed was analyzed by using a thermal conductivity detector (TCD).

The carbon formation and type on the used Ni/zCeyMgxZnAl catalyst were studied by using the O_2-temperature-programmed oxidation (O_2-TPO) technique (Autochem II Chemisorption analyzer: Micromeritics).In the experiment, the 10% O_2 in He gas with a total flow rate of 50 ml/min was fed to the system, and the system temperature raised to 1000°C using a heating rate of 5°C/min. The O_2-TPO profile was recorded by using a thermal conductivity detector (TCD).

1.3 In situ DRIFTS experimental

In situ DRIFTS was performed with a Bruker FTIR spectrometer equipped with a high sensitive mercury cadmium telluride (MCT) detector with a liquid-nitrogen-cooled system. For preparation before the IR studies, the catalyst (20 mg) was activated under H_2 atmosphere at 700°C for 1 h in a chamber and then the system was cooled down to room temperature with He gas. After that, the reduced catalyst was positioned inside the DRIFTS cell fitted with CaF_2 window. The CH_4 and CO_2 reactant gases with 40 ml/min of total flow rate at the 1:1 ratio was transferred to the cell and then heated to 450°C for 30 min (5°C/min) and recorded the spectrum every 10 min. Each spectrum was collected at 128 scans with an instrument resolution of 4 cm^{-1} over the 1000–4000 cm^{-1} spectrum range.

2. Results and discussion

Table S1 Textural properties of Ni/zCeyMgxZnAl catalyst.

Catalyst	Surface area (m^2/g)	Pore volume (cm^3/g)	Average pore diameter (nm)
Ni/CeMgAl	80	0.21	10.0
Ni/CeZnAl	34	0.14	15.6
Ni/CeMgZnAl	51	0.26	22.1
Ni/CeMg0.5ZnAl	48	0.18	13.0
Ni/Ce	11	0.06	18.4

Table S2 The fitting result of sp^2-carbon and sp^3-carbon hybridization, and the relative sp^2 /sp^3 intensity ratio evaluated from XPS measurements of used catalyst.

Catalyst	sp^2-carbon (%)	sp^3-carbon (%)	sp^2/sp^3
Ni/CeMgAl	48.3	21.8	2.22
Ni/CeZnAl	69.4	11.2	6.18
Ni/CeMgZnAl	68.9	14.6	4.73
Ni/CeMg0.5ZnAl	68.7	14.7	4.68
Ni/Ce	60.6	17.3	3.50

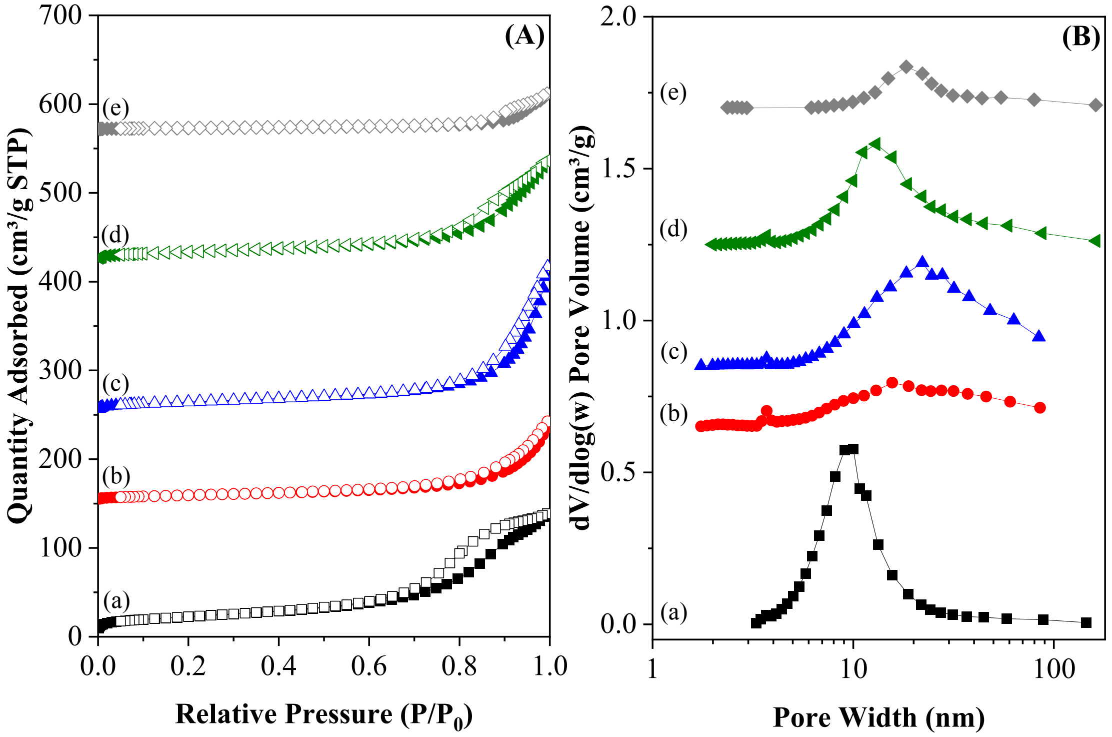

Figure S1 (A) N_2 adsorption-desorption isotherms and (B) pore size distributions of Ni/zCeyMgxZnAl catalysts. (a) Ni/CeMgAl, (b) Ni/CeZnAl, (c) Ni/CeMgZnAl, (d) Ni/CeMg0.5ZnAl, (e) Ni/Ce.

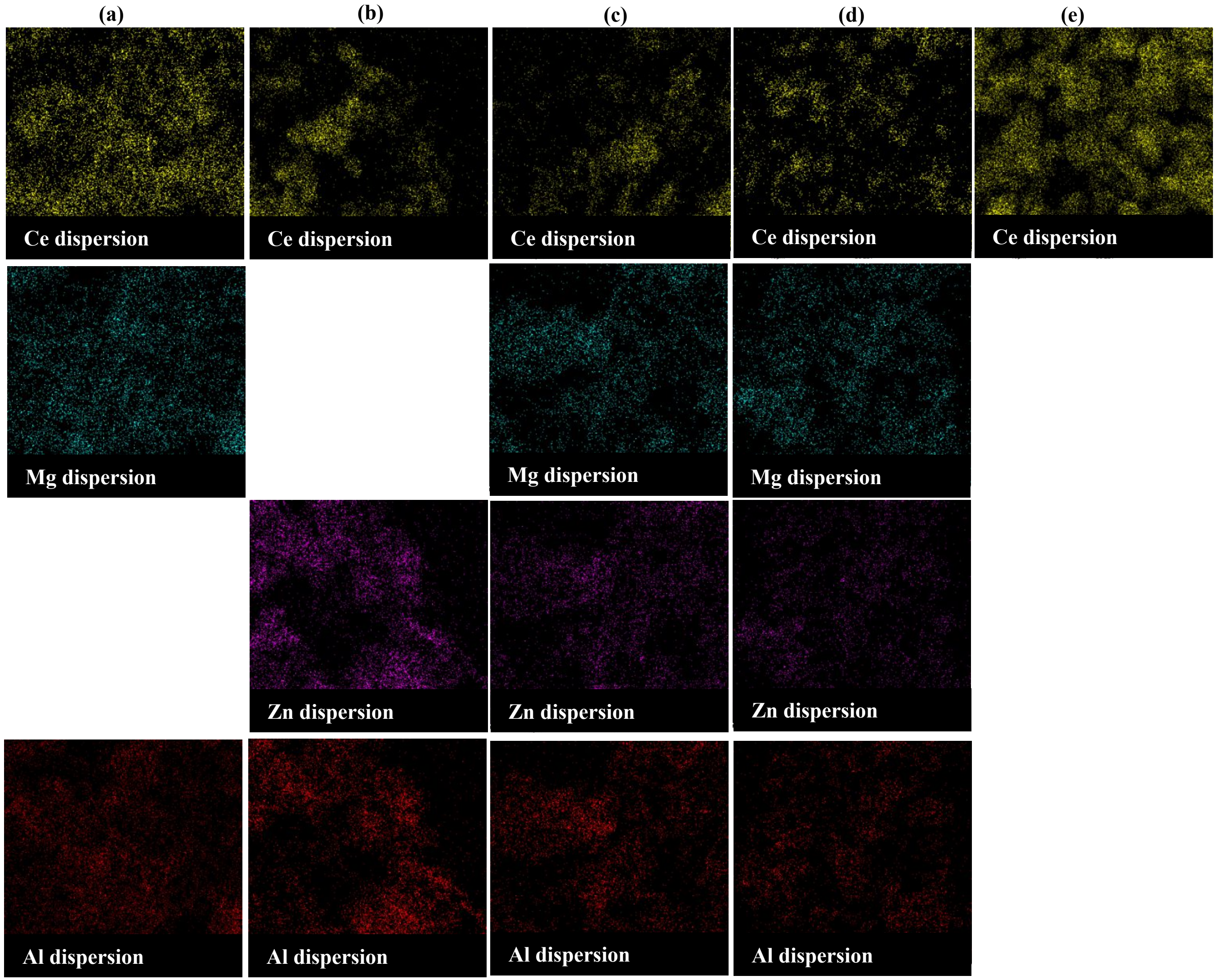

Figure S2 EDS-mapping of Ce, Mg, Zn, and Al of fresh catalysts. (a) Ni/CeMgAl, (b) Ni/CeZnAl, (c) Ni/CeMgZnAl, (d) Ni/CeMg0.5ZnAl, (e) Ni/Ce.
